# Hepatitis B in Ghana: a systematic review & meta-analysis of prevalence studies (1995-2015)

**DOI:** 10.1186/s12879-016-1467-5

**Published:** 2016-03-18

**Authors:** Richard Ofori-Asenso, Akosua Adom Agyeman

**Affiliations:** Research Unit, Health Policy Consult, P. O. Box WJ 537, Weija-Accra, Ghana

**Keywords:** Hepatitis B, Viral infections, Endemicity, Ghana, Meta-analysis

## Abstract

**Background:**

Although, chronic hepatitis B (HBV) is considered to be of significant public health importance in Ghana, not many reviews detailing the burden (prevalence) of the disease have been conducted. This study was aimed at summarizing the available information and to make an accurate estimate of HBV infection prevalence in Ghana over the last two decades (1995–2015).

**Methods:**

A systematic search was conducted in PubMed, ScienceDirect, Google Scholar and Africa Journals Online (AJOL) databases to retrieve primary studies published between 1^st^ January 1995 and 4^th^ October 2015, assessing the prevalence of HBV among populations in Ghana. This was supplemented by a manual search of retrieved references.

**Results:**

Thirty (30) studies across all the ten (10) regions of Ghana and involving an overall population size of 105,435 were analyzed. The national prevalence of HBV as determined by HBsAg seropositivity was 12.3 %. HBV prevalence among voluntary blood donors (VBDs), replacement blood donors (RBDs) and pregnant women were 10.8, 12.7 and 13.1 % respectively. HBV infection prevalence was highest among studies published within the period 1995–2002 (17.3 %), followed by those published within 2003–2009 (14.7 %) and the lowest prevalence rate being recorded across studies published in the period 2010–2015 (10.2 %). Regional prevalence were determined for Ashanti, Greater Accra, Eastern, Northern, central and Brong-Ahafo regions as 13.1, 10.6, 13.6, 13.1, 11.5 and 13.7 % respectively. No aggregate data were derived for Volta, Western, Upper East and Upper West regions. Higher prevalence of HBV infection was attained for rural (13.3 %) compared to urban settings (12.2 %). Across the country, highest HBV infection prevalence rates were recorded in persons within the age group 16–39 years.

**Conclusion:**

Hepatitis B infection is clearly an important public health problem in Ghana. The burden of the disease as dictated by a high prevalence rate calls for urgent public health interventions and strategic policy directions to controlling the disease to avert any potential future explosion.

## Background

Viral Hepatitis A, B, C, D and E cause significant morbidity and mortality affecting more people worldwide than even HIV [1]. Nearly 2 billion people across the world are estimated to be infected with Hepatitis B Virus (HBV) with nearly a quarter of this having chronic infection [2, 3]. Each year, over half a million HBV-related deaths are recorded across the globe [4].

Epidemiological studies have often demonstrated varying levels of endemicity of HBV worldwide, with highly endemic areas present in Sub-Saharan Africa (SSA) and East Asia, where between 5 and 10 % of the adult population are deemed to be chronically infected [5]. In Western Europe and North America, Less than 1 % of the population is chronically infected. The lifetime risk of infection from HBV in many African and Asian countries, the Amazon Basin and parts of the Middle East is estimated to be more than 60 % [6]. Accurate determination of the burden of HBV in Africa is difficult owing to poor record keeping and under-reporting, but estimates put that about 70–95 % of the adult population show evidence of past exposure to HBV infection and the HBsAg seroprevalence rate has been put around 6–20 % [6, 7].

Chronic HBV increases individuals’ risk of progressive liver disease and hepatocellular carcinoma (HCC). In the natural history of HBV infection, it is estimated that 10 to 33 % of those who develop persistent infection would end up with chronic hepatitis of which 20 to 50 % may develop liver cirrhosis [8]. HCC is a highly aggressive cancer with limited treatment options, often lacking in many resource-poor settings such as Africa [9]. SSA has one of the highest HBV-related liver cancer rates in the world [10]. HBV-related liver cancer also remains the most common cancer among males and the third most common cancer among females in the African region [11, 12]. Of significant importance is that, the average age of HCC development in Africa is considerably younger than in other more advanced regions (mean age 33 years compared to 60 years in Western Europe) meaning HBV-related HCC affects patients in their most productive and reproductive years [9]. HBV therefore represents a critical threat to health as well as other developmental and economic parameters on the African continent.

In Ghana, HBV is considered to be of significant public health importance and a disease that requires greater attention [13, 14]. Ghana has been grouped as part of the areas of the world where the prevalence of chronic HBV infection is high (≥8 %) [9, 15]. Sweitzer et al. for instance in estimating the global burden of hepatitis B in 2013, put the prevalence of chronic hepatitis B virus infection in Ghana at 12.92 % [3]. However, this was derived from an analysis of only 12 studies. Some experts have also put the prevalence rate of HBV in Ghana to be around 10–15 % [16, 17]. A scoping of the literature identified no other thoroughly conducted reviews specifically summarizing data on prevalence of HBV in Ghana. This observation points out that although there may have been significant research into understanding the burden of HBV in Ghana, the evidence available remains fragmented. To overcome this perceived gap in evidence compilation, we conducted a systematic review to thoroughly summarize the available information towards answering the key question; what has been the prevalence of HBV in Ghana over the last two decades (1995–2015)?

## Methods

### Study area

Ghana is located along the Gulf of Guinea and Atlantic Ocean, in the sub region of West Africa 4° north of the Equator. It has a land mass of 238,537 km^2^ and a population of approximately 27million of which 59 % is rural. Life expectancy at birth in 2013 was 62 years for males and 64 years for females [18]. The country is divided into ten administrative regions and 216 districts. Fifty-nine per cent of the Ghanaian workforce is in agriculture and almost everyone living in rural areas is involved in farming. Per capita total health expenditure as a percentage of GDP was 5.4 % in 2013 [18]. About 24.2 % of Ghanaians lived below the poverty line in 2013 [19]. In 2014, Ghana ranked 138 out of 187 countries and territories, with a Human Development Index value of 0.573 [20]. Fifty eight percent Ghanaians live less than 30 min of a public or private health facility, with better geographical within urban centres than rural settings [21]. The doctor-patient ratio in Ghana in 2011 was 1:10,034 [22] and almost 80 % of Pharmacists are based in the two largest cities Accra and Kumasi [23]. About 60 % of Ghanaians who report ill or injured consult a health practitioner while about one-third purchase medicines directly from retail drug outlets [21].

### Search strategy

To identify relevant studies, we conducted a structured review of the literature followed by an analysis of the reported prevalence rates. The review was conducted in accordance with the PRISMA (Preferred Reporting Items for Systematic Reviews and Meta-Analyses) Statement [24]. A comprehensive search was conducted in PubMed, Science Direct, Google scholar, and Africa Journals Online (AJOL) databases. The key words used were Hepatitis B, Hepatitis B surface antigen, prevalence, Ghana, and similar terms such as HBV, HBsAg, were crossed. The main limits used were ‘Humans’ and ‘English’.

### Inclusion and exclusion of studies

We included only primary studies published in peer-reviewed Journals between 1^st^ January 1995 and 4^th^October 2015, which reported prevalence of HBV in a sample population in Ghana. Study quality was assessed using a 12-point scoring system based on the Downs and Black checklist as adopted in similar reviews [25, 26]. These were: (objective of the study clearly described, study design clearly stated, participants representative of the population from which they were recruited, participants accrued during the same time period, modest sample size, management of missing data, age, gender and other characteristics explored/reported, e.g. were confounders reported, was detection method of HBV reported, were potential biases reported, was outcome clearly described?), the assessment also included other items known to be associated with study quality [26]. Studies were graded as high, medium or low based on quality scores. As globally agreed, laboratory diagnosis of hepatitis B infection focuses on the detection of the hepatitis B surface antigen HBsAg [5, 27]. Studies were included only if they presented HBV prevalence based on HBsAg seropositivity. Each study was issued with a unique number for identification purposes and the following descriptive information collected; author details, year of publication, region of Ghana, type of study population, mean age of subjects, number of subjects involved (sample size), setting (rural or urban) and the HBV prevalence (as defined by HBsAg seropositivity). Study data were extracted by RO and Cross checked by AA. Where there were disagreements, these were resolved by consensus-based discussions.

### Data analysis

The studies’ results were analyzed by meta-analysis proportions which was performed with StatsDirect statistical software version (Version 3.0.0, StatsDirect Ltd, Cheshire UK) [28]. Individual study proportions were assessed at 95 % confidence interval (CI) as well as the pooled effect. Test for heterogeneity was performed for all the proportions based on Cohran’s Q and degree of inconsistency (*I*^2^) [29]. In all the summary or pooled analysis, random effect model was adopted over fixed effect model due to the presence of heterogeneity resulting from variations of effects from individual studies confirmed by I^2^ > 0 %. For all computations, statistical significance was set at *p* < 0.05. We conducted sub-analyses for the periods within which studies were published (1995–2002, 2003–2009 and 2010–2015), among diverse population sub-groups (e.g. voluntary blood donors, family replacement donors and pregnant women & parturients) and across the various regions of Ghana.

### Ethical approval

This study did not require an ethical approval as it was based on information/data retrieved from published studies already available in the public domain.

## Results

### Studies identification and retrieval

**Fig. 1:**
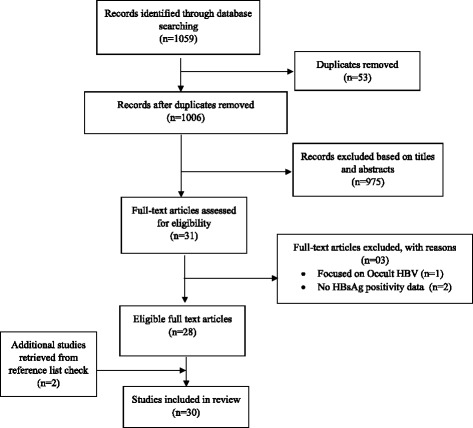
Schematic flow diagram of studies’ retrieval process

Figure 1 outlines the schematic flow of the studies identification and inclusion processes. A total of 1,059 articles were identified by literature search. After the exclusion of duplicates and irrelevant studies based on titles and abstracts, 31 articles were retrieved for detailed full-text analysis. Out of the 30 studies, 28 met the inclusion criteria for addition to the review. Two (2) additional studies were retrieved from the reference screening bringing the total number of studies included in the review to 30 [8, 30–58]. The 30 studies (Table 1) included in the review reported prevalence based on an overall population sample size of 105,435 across all the ten (10) regions of Ghana. The regional distribution of studies were, Ashanti (15), Greater Accra (5), Northern (2), Brong-Ahafo (2), Central (1), Eastern (1), Upper East (1), two (2) inter-regional studies as well as one (1) national study involving all 10 regions. About 77 % (23/30) of the studies were published in the years 2006–2015 compared to 23 % (7/30) for the period 1995–2005. A significant proportion of the studies (87 %, 26/30) were conducted in urban settings. Also, about 60 % (18/30) of the studies were published within the last 5 years (2010–2015) of the review period. Across studies, the sample sizes ranged from 110 to 51,100. About 47 % (14/30) of the studies were conducted in blood donors (voluntary and replacement family members). Only 20 % (6/30) of studies were conducted exclusively in disease-specific patient groups (type-2 diabetes, HIV, Sickle cell anemia and cirrhosis). The overall quality grading identified 57, 33 and 10 % of studies to be of high, moderate and low quality respectively.

**Table 1:** A summary of the descriptive characteristics of included studies

Study No	Author Details	Year of Publication	Design	Region of study	Study population	Age group	Setting	Sample size(n)	Method	HBsAg + (%)	Quality Grade
1	Addai-Mensah et al. [30]	2015	Cross-sectional	Ashanti	Blood donors^b^	17–60	Urban	400	Rapid test	6.75 %	Medium
2	Adjei et al. [31]	2006	Cross-sectional	Eastern & Greater Accra	Prison inmates & Officers	17–84	Urban	363	ELISA	14.3 %	High
3	Adjei et al. [32]	2008	Cross-sectional	National (Excludes Upper East & West)	Prison inmates & Officers	16–84	Urban	1811	ELISA	22.1 %	High
4	Allain et al. [33]	2003	Cross-sectional	Ashanti	Blood donors & Patients	16–59	Urban	383	Mixed methods	13.85 %	High
5	Allain et al. [34]	2009	Cross-Sectional	Ashanti	Blood donors	>16 years	Urban	51100	Rapid test	11.3 %	High
6	Allain et al. [35]	2010	Cross-sectional	Ashanti	Blood donors	31.0^a^	Urban	11000	Rapid test	14.2 %	High
7	Amidu et al. [36]	2010	Cross-sectional	Upper East	Blood donors	17–58	Urban	4146	Rapid test	12.64 %	Medium
8	Amidu et a.l [37]	2012	Cross sectional	Ashanti	Community screenees	15–38	Urban	783	Rapid test	8.68 %	High
9	Antwi-Baffour et al. [38]	2014	Cross sectional	Greater Accra	Sickle cell patients	n.s	Urban	202	Rapid test	3.50 %	Low
10	Apea-Kubi et al. [39]	2006	Cross-sectional	Greater Accra	Pregnant & non-pregnant women	29.6^a^	Urban	517	Rapid test	16.8 %	High
11	Blankson et al. [8]	2005	Case-control	Greater Accra	Cirrhotic & non-cirrhotic patients	15–90	Urban	350	ELISA	14.6 %	High
12	Candotti et al. [40]	2007	Cross-sectional	Ashanti	Pregnant women	n.s	Urban	1368	Rapid test	16.0 %	High
13	Cho et al. [41]	2012	Cross-sectional	Eastern	Pregnant Women	n.s	Urban	1500	Rapid test	10.6 %	High
14	Damale et al. [42]	2005	Cross-sectional	Greater Accra	Parturients (pregnant women)	27.0^a^	Urban	638	n.s	10.5 %	Medium
15	Dongdem et al. [43]	2012	Cross-sectional	Northern	Blood donors	20–29 years	Urban	6321	Rapid test	11.5 %	High
16	Ephraim et al. [44]	2014	Cross-sectional	Central	Type 2 Diabetics	n.s	Urban	110	Rapid test	5.50 %	Medium
17	Ephraim et al. [45]	2015	Cross-sectional	Ashanti	Pregnant women	10–40 year	Urban	168	Rapid test	16.0 %	Medium
18	Geretti et al. [46]	2010	Cross sectional	Ashanti	HIV patients	n.s	Urban	838	Mixed methods	16.7 %	High
19	Kubio et al. [47]	2012	Report review	Northern	Blood donors	n.s	Rural	853	n.s	7.50 %	Low
20	Matinson et al. [48]	1996	Cross-sectional	Ashanti	Children	6–18	Rural	803	immunoassay	15.8 %	Medium
21	Matinson et al. [49]	1998	Cross-sectional	Ashanti	Community screenees	. > 1	Rural	1385	immunoassay	20.9 %	Medium
22	Mutocheluh et al. [50]	2014	Cross-sectional	Brong-Ahafo	Blood donors	17–57	Urban	164	ELISA	14.6 %	High
23	Nkrumah et al. [51]	2011	Cross-sectional	Ashanti	Blood donors	26–35 years	Rural	2773	Rapid test	10.53 %	Medium
24	Nsiah et al. [52]	2012	Cross-Sectional	Ashanti	Sickle cell patients	10–18	Urban	330	Immunoassay	3.60 %	Low
25	Owiredu et al. [53]	2012	Cross-sectional	National (all regions)	Blood donors	n.s	Urban	480	Mixed methods	8.13 %	High
26	Owusu-Ofori et al. [54]	2005	Cross-sectional	Ashanti	Blood donors	n.s	Urban	9372	Rapid test	13.4 %	High
27	Rufai et al. [55]	2014	Cross-sectional	Ashanti	Blood donors	16–59	Urban	150	Immunoassay	13.3 %	Medium
28	Sagoe et al. [56]	2012	Cross-sectional	Greater Accra	HIV patients	≥18 years	Urban	138	ELISA	13.0 %	High
29	Sarkodie et al. [57]	2001	Cross-sectional	Ashanti	Blood donors	16–52	Urban	3587	Mixed methods	15.3 %	High
30	Walana et al. [58]	2014	Cross-sectional	Brong-Ahafo	Blood donors	20–49 years	Urban	3402	ICT	9.60 %	Medium

^a^ average, *HBsAg* Hepatitis B surface antigen, *n.s* not specified, *VBD* Voluntary blood donor, *ELISA* Enzyme-Linked Immunosorbent assay, *ICT* immunochromatography, ^b^Blood donors is used to represent either voluntary or replacement donors or both

### Overall national prevalence

**Fig. 2:**
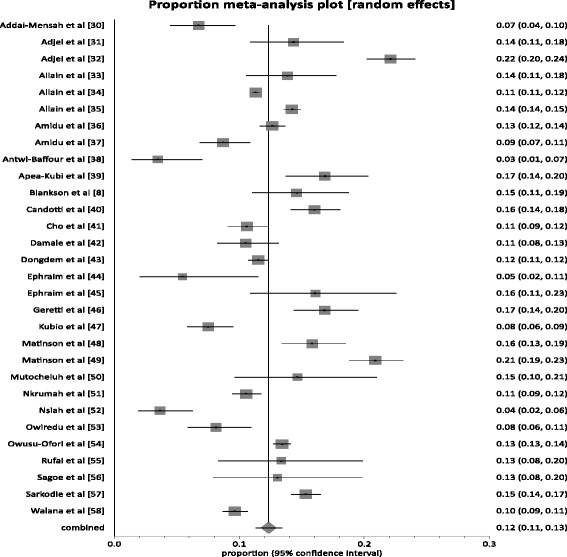
Forest plot of HBV infection prevalence rates in Ghana for studies published from 1995 to 2015

Of the 30 studies, the reported prevalence rates ranged from 3.5 to 22.1 % (Table 1). In 83 % (25/30) of studies, the reported prevalence rates exceeded 8 %; the level used to define the endemicity level of an area as high. In 20 % (6/30) of studies, the reported prevalence was at least twice the 8 %. The pooled prevalence rate (Fig. 2) nationally across the 30 studies was 12.3 % (95 % CI 11.3 to 13.4; *P* < 0.0001). The result of heterogeneity was also 94.6 % (95 % CI 93.6 to 95.4 %) for the degree of inconsistency.

**Fig. 3:**
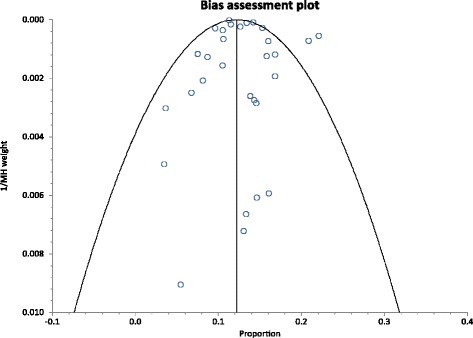
Bias Assessment plot of studies reporting HBV prevalence in Ghana from 1995 to 2015

There was no evidence of publication bias with Egger’s test having a *P* = 0.5273, whereas Begg’s test had a *P* = 00.8045. This was depicted graphically by a funnel plot which showed a near symmetrical display of prevalence reported by various studies (Fig. 3).

### Blood donors

**Fig. 4:**
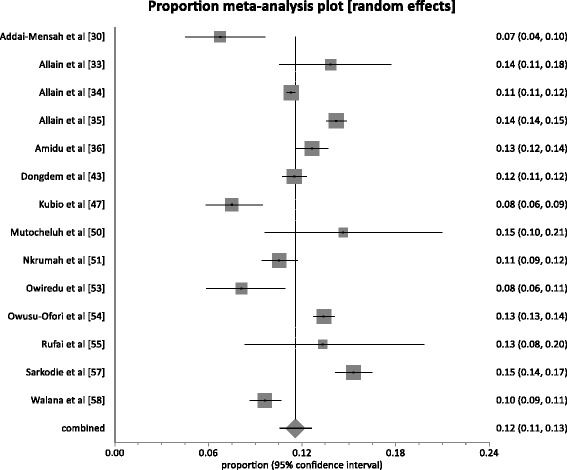
Forest plot of HBV infection prevalence rates among blood donors (VBDs & RBDs) in Ghana

Fourteen studies comprising a total population sample size of 93,990 presented HBV prevalence among blood donors (Voluntary and replacement blood donors). The HBV prevalence rate among these blood donors (BDs) as presented by these studies was within the range 6.75–15.3 %. For the 14 studies, 86 % (12/14) reported prevalence rates above 8 %, the level used to define the endemicity level in an area as high [7]. The pooled prevalence rate (Fig. 4) across the 14 studies was 11.6 % (95 % CI 10.6 to 12.6; *P* < 0.0001). The result of heterogeneity was also 93.2 % (95 % CI 90.8–94.8 %) for the degree of inconsistency. Of the 14 studies on blood donors, 7 studies provided individual prevalence on voluntary blood donors (VBDs) and replacement donors (RBDs) based on a total sample size of 80,709 (VBDs = 44,213, RBDs = 36,496). Among these studies, the pooled HBV prevalence among VBDs was 10.8 % (95 % 8.6 to 13.2; *P* < 0.0001) whereas among RBDs the prevalence rate was 12.7 % (95 % 11.1 to 14.4; *P* < 0.0001). The difference (1.90 %; 95 % CI = 1. 5 to 2.4 %) in HBV prevalence rate between the two donor types was statistically significant (*P* < 0.0001).

### Pregnant women & parturients

**Fig. 5:**
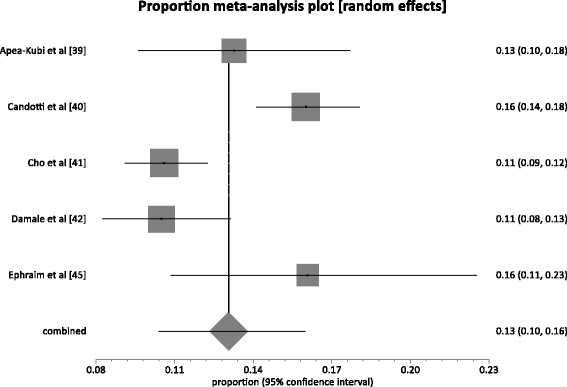
Forest plot of HBV infection prevalence rates among pregnant women and parturients in Ghana

A total of 5 studies presented HBV prevalence rates among pregnant women and parturients. The combined sample size across these studies was 3,968. The HBV prevalence rate in this group ranged from 10.5–16.0 %. The pooled prevalence rate (Fig. 5) across the 5 studies was 13.1 % (95 % CI = 10.4 to 16.0 %; *P* < 0.0001). The result of heterogeneity was also 82.9 % (95 % CI = 51.2 to 90.9 %) for the degree of inconsistency. The difference in prevalence rate (1.5 %) between the pregnant & parturient women population and the general population was not statistically significant (*P* = 0.1324).

### Prevalence related to socio-demographic characteristics

Across studies included in the review, there was remarkable diversity in participants’ age groups, although, majority of the studies were conducted in adult populations (>16 years). The most prevalent age groups were not specified in 15 studies [31, 34, 40, 44–47, 50–54, 56, 57]. One study reported the highest prevalence of HBV infection in participants aged over 40 [33], while 9 studies reported in those aged 16–39 years. In 3 studies, the most prevalent HBV participants were aged ≥20 years [49, 55. 58]. In one (1) study the most prevalent HBV participant population was <16 years [49] where as in two (2) studies no observable difference was observed among different age groups [41, 48]. The gender of the participants was not specified in 5 studies [46, 47, 52–54]. Five studies restricted to females [39–42, 45] and no study restricted to males only. Two studies [48, 51] documented no difference among male and female participants. Five (5) studies reported higher HBV prevalence among male participants in comparison with females [30, 36, 37, 49, 58] while opposite results were reported in two studies [32, 38]. One national study reported higher infection rate in females than males [32]. Two studies reported higher prevalence in married participants than unmarried persons [42, 50] whereas one study reported opposite results [32]. Two studies documented history of previous blood donation and one indicated higher infection in persons with previous history of blood donation [32] while the other study demonstrated no difference with blood donation non-recipients [50]. One study reported higher prevalence in homosexuals compared to heterosexuals [32].

### Prevalence comparison in rural and urban settings

**Fig. 6:**
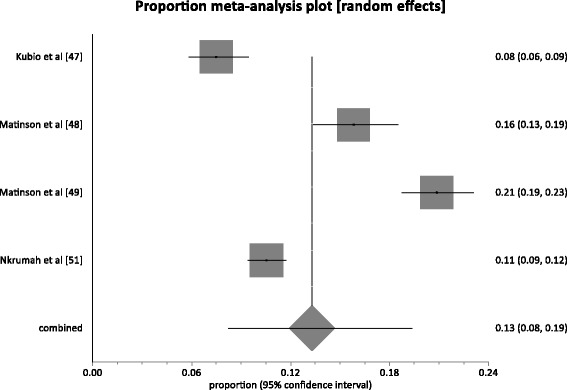
Forest plot of HBV prevalence rates for studies conducted in rural settings in Ghana

**Fig. 7:**
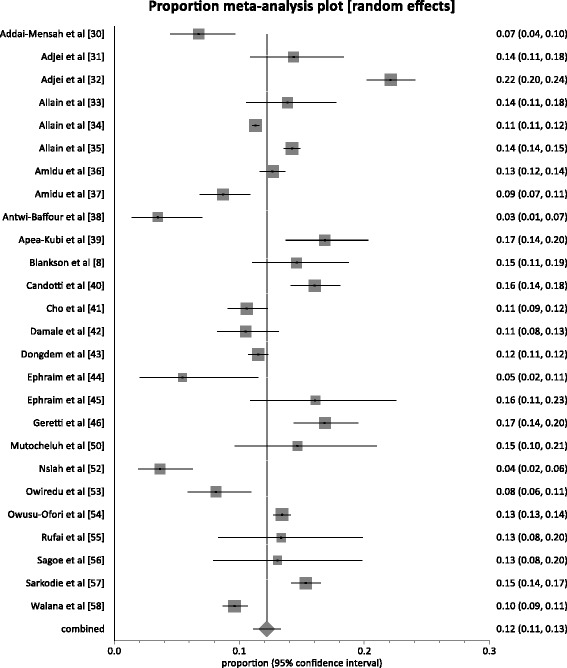
Forest plot of HBV prevalence rates for studies conducted in urban settings in Ghana

Four (4) studies were conducted within rural settings. These studies presented HBV based on a combined sample size of 5,814. The pooled prevalence rate (Fig. 6) across the studies conducted in rural settings was 13.3 % (95 % CI 8.2 to 19.4; *P* < 0.0001). The result of heterogeneity was also 97.3 % (95 % CI = 95.9 to 98.1 %). Twenty-six (26) studies were conducted within urban settings. These studies presented HBV based on a combined sample size of 99,621. The pooled prevalence rate (Fig. 7) across the studies conducted in urban settings was 12.2 % (95 % CI = 11.1 to 13.3; *P* < 0.0001). The result of heterogeneity was also 94.1 % (95 % CI = 92.8 to 95.0 %). The difference (1.1 %; 95 % CI = 0.21 to 2.02 %) in HBV prevalence rates across studies conducted in urban setting and those in rural settings was statistically significant (*P* = 0.0129).

### HBV prevalence by region

**Fig. 8:**
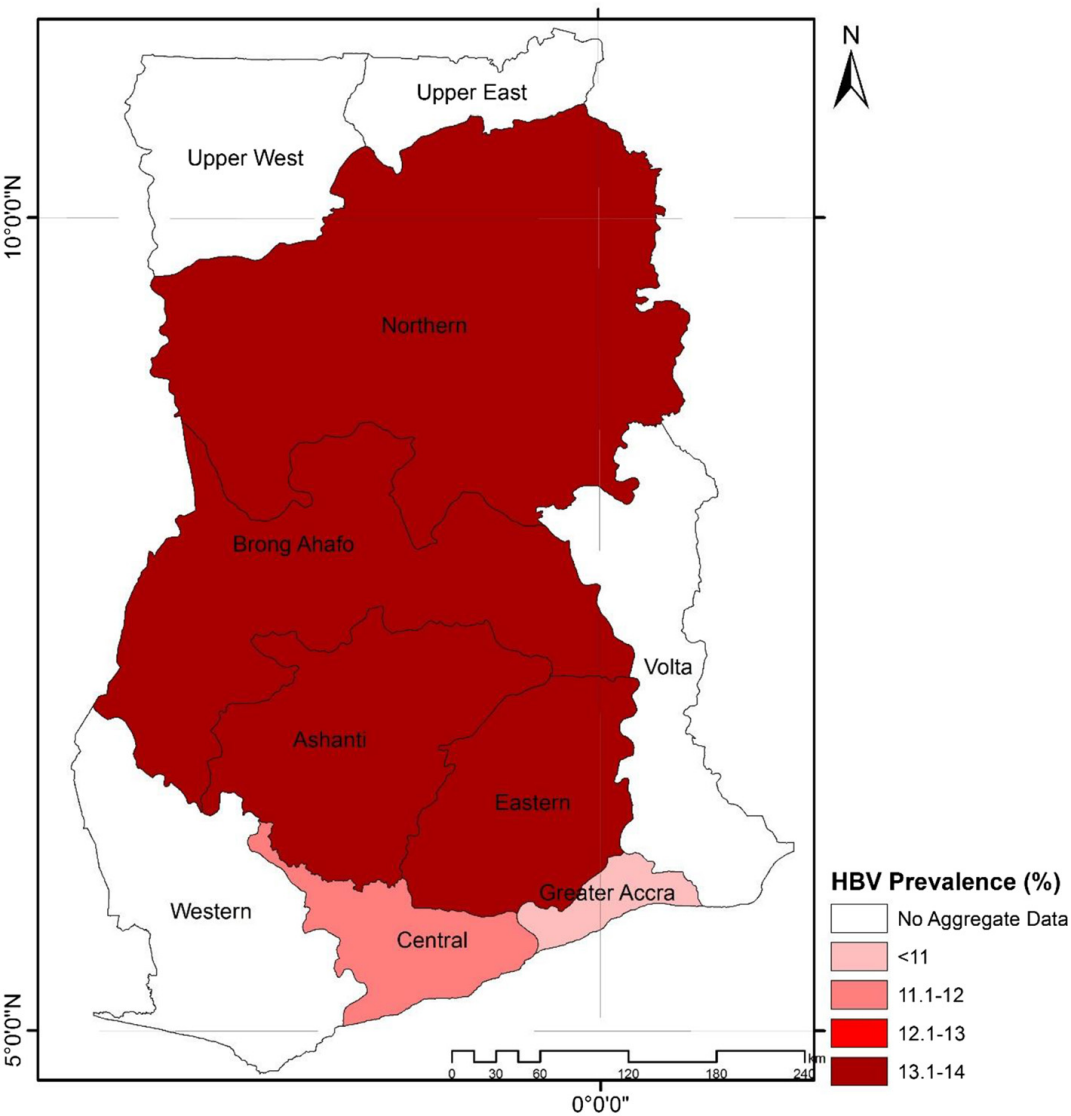
Map of HBV prevalence across different regions in Ghana

Eight (8) studies presented HBV prevalence rates for the Greater Accra region, the second most populous region in Ghana. Among these studies, the HBV prevalence rates ranged from 0 to 16.8 %. The studies presented HBV prevalence based on a total population of 2,416. The pooled prevalence rate across the eight (8) studies was 10.6 % (95 % CI = 7.3 to 14.3 %; *P* < 0.0001). The result of heterogeneity was also 86.5 % for the degree of inconsistency. Five (5) studies presented HBV prevalence rates for the Brong-Ahafo region. Among these studies, the HBV prevalence rates ranged from 6.0 to 24.5 %. The studies presented HBV prevalence based on a total population of 3,828. The pooled prevalence rate across the 4 studies was 13.7 % (95 % CI = 7.1 to 22.1 %; *P* < 0.0001). The result of heterogeneity was also 91.92 % for the degree of inconsistency. Four (4) studies presented HBV prevalence rates for the Eastern region. Among these studies, the HBV prevalence rates ranged from 10 to 20 %. The studies presented HBV prevalence based on a total population of 1,766. The pooled prevalence rate across the 4 studies was 13.6 % (95 % CI = 9.3 to 18.5 %; *P* < 0.0001). The result of heterogeneity was also 64.3 % for the degree of inconsistency. Three (3) Studies presented HBV prevalence rates for the central region, with prevalence rates ranging from 5.5 to 22.8 % based on a sample size of 796. The pooled prevalence rate across the 3 studies was 11.5 % (95 % CI = 3.9 to 22.5 %; *P* < 0.0001). The result of heterogeneity was also 93.28 % for the degree of inconsistency. Prevalence data for the Northern region, the largest region in Ghana was retrieved from four (4) studies. Among these studies, the HBV prevalence rates ranged from 7.5 to 24.7 %. The studies presented HBV prevalence based on a total population of 7,488. The pooled prevalence rate across the 4 studies was 13.1 % (95 % CI = 8.6 % to 19.8; *P* < 0.0001). The result of heterogeneity was also 93.93 % for the degree of inconsistency. For Ashanti region, the most populous region in Ghana, prevalence data were retrieved from seventeen (17) studies comprising a combined sample size of 84,694. The HBV prevalence rate for this region ranged from 3.6 to 20.9 %. The pooled prevalence rate across the 17 studies was 13.1 % (95 % CI 11.7 to 14.5; *P* < 0.0001). The result of heterogeneity was also 94.9 % for the degree of inconsistency. For the Volta, Upper East, Upper West and Western regions, it was not possible to generate aggregate data for the Volta, Upper east, upper west and western regions (Fig. 8).

### Prevalence by studies’ publication period

**Fig. 9:**
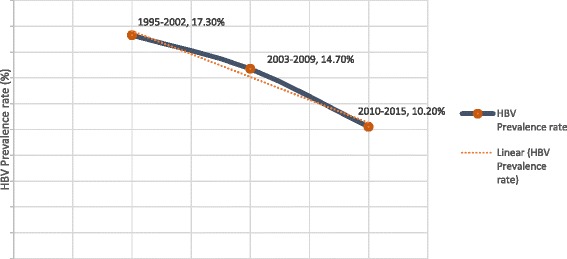
A graph of HBV infection prevalence in Ghana according to studies’ publication periods

Studies’ publication periods were grouped into 1995–2002, 2003–2009 and 2010–2015. Three (3), Nine (9) and eighteen (18) studies were published within these periods respectively. Among the studies published in the period 1995–2002, the HBV prevalence ranged from 15.3 to 20.9 % based on a combined sample size of 5,775. The pooled prevalence rate across the studies published within the period 1995–2002 was 17.3 % (95 % CI 13.8 to 21.0; *P* < 0.0001). The result of heterogeneity was also 90.83 %. The nine (9) studies published within the period 2003–2009 also presented HBV prevalence data based on a combined population size of 65,902. The HBV prevalence rates for studies published within this period ranged from 10.5 to 22.1 %. The pooled prevalence rate across the studies published within the period 2003–2009 was 14.7 % (95 % CI 12.5 to 17.0; *P* < 0.0001). The result of heterogeneity was also 96.19 %. For the 18 studies published within the last 5 years (2010–2015) preceding the conduct of this review, the HBV prevalence ranged from 3.6 to 16.8 % based on a combined sample size of 33,758. The pooled prevalence rate (Fig. 6) across the studies published within the period 2010–2015 was 10.2 % (95 % CI 8.9 to 11.6; *P* < 0.0001). The result of heterogeneity was also 91.78 %. Hence, the ascending order of prevalence according to studies’ publication period was 2010–2015 < 2003–2009 < 1995–2002 (Fig. 9).

## Discussion

Hepatitis B virus (HBV) infection is a major problem of public health in the world particularly in developing countries. Our review identified the prevalence of HBV in Ghana as detected by HBsAg seropositivity to be high at 12.3 %. Our result is comparable to the prevalence rate reported by Sweitzer et al‘s. review [3] albeit larger number of studies involved in our study. The results achieved is also in alignment with the categorization of Ghana as a high HBV endemic country (prevalence ≥8 %) [8, 15]. The prevalence rate achieved significantly exceeds the reported global prevalence rate of 3.61 % as well as the rate of 8 · 83 % for the WHO Africa region [3]. In countries like the US, HBV prevalence rate has been estimated to be around <0.27 % [59]. In Iran and Kosovo current estimates put HBV prevalence at 2.14 and 4.2 % respectively [60, 61]. Such comparative information further highlight the enormity of the HBV burden in Ghana.

Our study also raises serious concerns regarding the safety of blood supply in Ghana as nearly 1 in 9 blood donors may be infected with HBV with even higher proportions in replacement blood donors. Ghana has a national blood policy which requires the screening of all donated blood for HIV 1 and 2, HBV, Hepatitis C and Syphilis [62]. Our findings however highlight the need for stricter adherence to such policies as the risk of receiving contaminated blood, which in this is HBV remains high. Additionally, HBV infection among pregnant women also remains high (≈1 in 8) and which justifies the establishment of a national HBV screening program for all pregnant women in antenatal clinics throughout Ghana. Additionally, a national policy to vaccinate all pregnant women who test negative for HBV should be adopted so as to reduce the risk of mother to child transmission within the population [45].

A number of factors may account for the observed high HBV prevalence in Ghana. This includes lack of adequate information and understanding among Ghanaians of the transmission dynamics of the virus. For instance, in an assessment of 200 barbershops within the Kumasi metropolis, only 7 % knew the route of transmission of HBV [63]. Akumiah and Sarfo further point out that, the barber community in Ghana paid more attention to the decoration (e.g., availability of television, air conditioning, sound system etc.) other than the risk factors associated with their profession in the transmission of diseases such as HBV [64]. Although, the 3 main transmission routes of HBV in Ghana are transfusion of infected blood, unprotected sex and mother to child transmission, and most Ghanaians with chronic hepatitis B were infected at birth or in childhood, HBV has often been framed as a sexually transmitted infection in many communities and even among health workers [13]. Stigmatization arising from such misconceptions has many times prevented patients from finding their way to proper care and subsequently reducing their infectivity rates.

There are three (3) key components to controlling hepatitis B. These include treating infected persons, interrupting the spread of the infection transmission and reducing the mortality associated with advanced hepatic disease and HCC [65]. A vaccine against hepatitis B has been available since 1982. The vaccine is safe and 95 % effective in preventing infection and the development of chronic disease and HCC due hepatitis B [5]. For instance, in Senegal, vaccinations have reduced infection rates among children from 18.7 to 2.2 %, whereas in Gambia, it has led to a reduction in infection rate from 10 % to less than 1 % [66]. Ghana introduced Hepatitis B vaccination of babies as part of the Expanded Programme of Immunization (EPI) in 2002 [13]. Babies from 6 weeks onwards receive the pentavalent vaccine (diphtheria, polio, tetanus, hepatitis B, influenza type B). The coverage of EPI is good in all regions of the country and among the highest in Sub-Saharan Africa [67]. The introduction of the HBV vaccine in 2002 may have by inference contributed to the lowering of prevalence rates as studies published in the post vaccine introduction periods 2003–2015 recorded lower HBV prevalence rates than the pre-vaccine introduction period (1995–2002).

On the other hand, although, Ghana’s National Health Insurance Scheme (NHIS) introduced in 2003, aims to improve access to health services by eliminating financial barriers (particularly out-of pocket payments), hepatitis B screening and vaccination in Ghana outside EPI are still not covered under the scheme. Screenings are only covered and prescribed at hospitals for patients suspected to be reactive to hepatitis B and/or C. Hepatitis B immunoglobulin G and hepatitis B monovalent vaccine for babies born to hepatitis B reactive mothers are also not covered by the NHIS [68]. These may have all hampered effective control of the disease over the last couple of years.

Epidemiological studies have demonstrated that rapid urbanization, overpopulated cities and poor socioeconomic conditions such as lack of access to clean water and sanitation are implicated in the burden of HBV [69]. The World Bank notes that over the last 2 decades, there has been a steady increase in the proportion of Ghanaians with access to portable water with current rates exceeding 88 % [70]. Nevertheless, less than 15 % of Ghanaians have access to proper sanitation [71]. Martinson et al. [48], has demonstrated that the improvement of socioeconomic conditions may lead to a decreasing exposure to viral hepatitis such as HBV in Ghana. Hence, the apparent reported lower prevalence rate within the period 2003–2015, may have been due to the combined effect of vaccine introduction and improvement in some socioeconomic conditions. Better socioeconomic improvement and vaccination coverage in urban areas compared to rural areas may underline the difference in HBV prevalence rates difference between these two settings. However, it is unclear the extent to which factors such as vaccination and socioeconomic conditions have played in the slight regional variations in HBV prevalence across the country.

Although, there exist significant gaps in the evidence documenting the burden of HBV on individuals, the healthcare system and the country as whole, the cost associated with HBV in Ghana can be enormous because of the high morbidity and mortality associated with end-stage liver disease, cirrhosis and HCC. Blankson et al., identified that over 2 in 5 cirrhotic patients in Ghana had chronic HBV [8]. The cost of oral treatment for HBV in Ghana is about GHC 300-400 (USD100-150) a month or same weekly to take an injection for 48 weeks as a way of managing the condition [72]. This cost is enormous and one that majority of Ghanaians cannot afford. Even if this was to be publicly funded, the impact on health expenditure would be significant. Moreover, as identified from the studies, the most prevalent population was 16–39 years covering some of the most productive age groups. Thus the economic impact of HBV in Ghana through loss of life and absenteeism from work cannot be underestimated.

Addressing Ghana’s high HBV prevalence should remain a key national priority and one that needs strategic public health interventions. In 2014, the World Health Assembly adopted the second WHO resolution on viral hepatitis (WHA67.6), providing guidance to governments on how to prioritize actions to tackle all forms of viral hepatitis in a coordinated manner [73]. Subsequently, the recently released WHO guidelines on the management of chronic hepatitis B highlights the importance of adopting a simplified public health approach to controlling the virus [74]. The key highlights of this guideline includes developing publicly-funded screening and treatment programmes and providing universal access to hepatitis B prevention, care and treatment. Scaling up this programme in Ghana will have two main benefits. Firstly, it will expand access to the general population. And secondly, it will strengthen the diagnostic services and laboratory infrastructure to support care. In line with this, Hepatitis B vaccination should be covered by the NHIS, preferably for every citizen. If this is not achievable owing to resource limitations, it should be made available at least to all family members/close contacts of persons with hepatitis B in efforts to reduce horizontal transmission of the disease.

Effectively tackling HBV burden calls for a stronger political will and a wider social involvement. The aim will be to solidify the inclusion of HBV prevention in the overall national health agenda and salvage the needed resources to execute the necessary interventions. Lemoine et al. makes interesting reference to lessons learnt from the HIV/AIDS epidemic and advocates that the same energy and mobilization must be applied to fighting viral hepatitis such as HBV [1]. Within the HIV/AIDS domain, pressure from patient advocacy groups and civil societies for instance “pushed” policy makers and drug manufacturers to lower the cost of ARTs to the current level of around USD100 per person per year from about $10,000 per patient per year in the early 2000s [1]. This has subsequently had tremendous impact on the number of individuals receiving ART. Also integrating viral hepatitis programmes into the existing national health programmes around Tuberculosis (TB) or HIV may allow shared synergies in terms of the programme’s success and limit its cost [1, 65].

This study raises a number of key issues regarding HBV research in Ghana. Firstly, while there seems to be a positive trend of increased research in this area characterized by 60 % of papers being published in the last 5 years, there are widespread regional variations in the level of research. About 75 % of studies were conducted in two regions (Ashanti & Greater Accra) and in four regions it was not possible to derive aggregate data due to limited number of studies. Moreover, studies were concentrated in adult populations giving little information about HBV prevalence in children which is a better predictor of the level of maternofaetal transmission [48]. Likewise most studies provided no information regarding the relationship between prevalence and socio-demographic characteristics such as religion, income, and type of occupation all of which are recognized predictors of HBV prevalence [75]. Further research in these areas would be needed to fully understand the dynamics of HBV burden in Ghana.

### Limitations

The accuracy of detection of active HBV infection depends on a number of factors such as the screening method employed [25]. The distinction of active from past infection usually requires the adoption of other methods including the identification of HBV IgM and HBV markers. Advanced methods such as DNA testing may be able to detect the presence of hepatitis even before the appearance of antibodies [25, 76]. Since, our review was conducted over a 20-year period, the studies involved may have used different generations of screening kits and there may be a variation in the sensitivity and specificity which could impact on the difference in prevalence rates between the different study publication periods. The majority of the studies analyzed used convenient samples and the risk profile may not be fully representative of the Ghanaian population. Moreover, of the 105,435 population size involved in the 30 studies, a significant proportion (89.1 % *n* = 93,990), were among blood donors who are populations with specific characteristics. In Ghana, women and children are not frequent donators of blood as compared to young and middle-aged men. Hence, the HBV prevalence, may be more representative of a young and middle-aged male population. Moreover, with the regional imbalance in the number of studies, the overall prevalence may not be entirely representative of a true national prevalence. Regardless of the above limitations, our review provides a useful estimate of the prevalence of HBV in Ghana as it is similar to prevalence rates quoted by some experts and also comparable to results from other countries within the sub-region [3, 16, 17]. Also, with the majority of studies published recently, the overall prevalence is likely to reflect current situation.

## Conclusion

In conclusion, there is a high burden of chronic HBV infection among Ghanaians. This is a preventable disease and indeed one that can be eradicated by vaccination. Addressing the challenges of HBV infection in Ghana will require multi-level approaches that includes population-wide educational interventions and a strengthening of the Ghanaian health system. There is urgent need for the government of Ghana and its international partners to prioritize HBV control as done successfully for conditions such as HIV, Malaria and TB. This is necessary to prevent a potential future explosion of HBV in Ghana.

## Availability of data and materials

We declare that the data supporting the conclusions of this article are fully described within the article.

## Abbreviations

AIDS: Acquired immune deficiency syndrome
ART: Antiretroviral therapy
HBV: Hepatitis B virus
HBsAg: Hepatitis B surface antigen
HCC: Hepatocellular carcinoma
HIV: Human immunodeficiency virus
NHIS: National health insurance scheme
PRISMA: Preferred reporting items for systematic reviews and meta-analyses
RBD: Replacement blood donor
SSA: Sub-Saharan Africa
TB: Tuberculosis
VBD: Voluntary blood donor
WHO: World Health Organization
ELISA: Enzyme-linked immunosorbent assay
ICT: Immunochromatography

## References

[1] Lemoine M, Eholié S, Lacombe K (2015). Reducing the neglected burden of viral hepatitis in Africa: strategies for a global approach. J Hepatol.

[2] Lok AS (2002). Chronic hepatitis B. New Engl J Med.

[3] Schweitzer A, HorJ J, Mikolajczyk RT, Krause G, Ott JJ (2015). Estimations of worldwide prevalence of chronic hepatitis B virus infection: a systematic review of data published between 1965 and 2013. Lancet.

[4] Goldstein ST (2005). A mathematical model to estimate to estimate global hepatitis B disease burden and vaccination impact. Int J Epidemiol.

[5] World Health Organization. Hepatitis B. http://www.who.int/mediacentre/factsheets/fs204/en/(2015). Accessed 01 Oct 2015.

[6] Ott JJ, Stevens GA, Groeger J, Wiersma ST (2012). Global epidemiology of hepatitis B virus infection: new estimates of age-specific HBsAg seroprevalence and endemicity. Vaccine.

[7] Kiire CF (1996). The epidemiology and prophylaxis of hepatitis B in sub-Saharan Africa: a view from tropical and subtropical Africa. Gut.

[8] Blankson A, Wiredu EK, Adjei A, Tettey Y (2005). Seroprevalence of hepatitis B and C viruses in cirrhosis of the liver in Accra, Ghana. Ghana Med J.

[9] Howell J, Ladep NG, Lemoine M, Thursz MR, Taylor-Robinson SD (2014). Hepatitis B in Sub-Saharan Africa. South Sudan Med J.

[10] Parkin DM, Bray F, Ferlay J, Pisani P (2005). Global cancer statistics. 2002.. CA Cancer J Clin.

[11] Parkin DM, Sitas F, Chirenje M, Stein L, Abratt R, Wabinga H (2008). Part I: Cancer in Indigenous Africans--burden, distribution, and trends. Lancet Oncol.

[12] Kirk GD, Lesi OA, Mendy M, Akano AO, Sam O, Goedert JJ (2004). The Gambia Liver Cancer Study: Infection with hepatitis B and C and the risk of hepatocellular carcinoma in West Africa. Hepatology.

[13] Owusu-Ansah T. Viral Hepatitis in Ghana: The Role of the Government http://www.ghanaweb.com/GhanaHomePage/NewsArchive/Viral-Hepatitis-In-Ghana-The-Role-Of-The-Government-222118. (2014). Accessed 01 Oct 2015.

[14] Mkandawire P, Richmond C, Dixon J, Luginaah IN, Tobias J (2013). Hepatitis B in Ghana’s upper west region: a hidden epidemic in need of national policy attention. Health Place.

[15] Averhoff F. Hepatitis B. http://wwwnc.cdc.gov/travel/yellowbook/2016/infectious-diseases-related-to-travel/hepatitis-b (2015). Accessed 02 Oct 2015.

[16] GhanaWeb. Ghana Rated High Risk for Hepatitis B and C. http://www.ghanaweb.com/GhanaHomePage/health/Ghana-rated-high-risk-for-Hepatitis-B-C-280781 (2013). Accessed 30 September 2015.

[17] Teye J. Ghana risks losing productive youth to Hepatitis-Medical Professor. http://www.myjoyonline.com/lifestyle/2015/September-15th/ghana-risks-losing-productive-youth-to-hepatitis-medical-professor.php (2015). Accessed 30 November 2015.

[18] World Health Organization. Ghana. http://www.who.int/countries/gha/en/(2015). Accessed 30 November 2015.

[19] Central Intelligence Agency. The World Factbook. https://www.cia.gov/library/publications/the-world-factbook/fields/2046.html (2014). Accessed 30 November 2015.

[20] UNDP. Ghana Launches the 2014 Human Development Report. http://www.gh.undp.org/content/ghana/en/home/presscenter/articles/2014/08/13/ghana-launches-the-2014-human-development-report.html (2014). Accessed 01 Oct 2015.

[21] Bosu WK (2010). Epidemic of Hypertension in Ghana: a systematic review. BMC Public Health.

[22] Ghana News Agency. Access to Pharmaceutical Services in favour of Urban Areas. http://www.ghananewsagency.org/health/access-to-pharmaceutical-services-in-favour-of-urban-areas-mr-nyoagbe-15819 (2010). Accessed 02 Oct 2015.

[23] Alhassan RK, Nicole Spieker N, Ostenberg PV, Ogink A, Nketiah-Amponsah E, Tobias F, Rinke de Wit TF. Association between health worker motivation and healthcare quality efforts in Ghana. Hum Resour Health. 2013;11:37. 10.1186/1478-4491-11-37 PMC3765920 23945073

[24] Moher D, Liberati A, Tetzlaff J, Altman DG (2009). Preferred reporting items for systematic reviews and meta-analyses: the PRISMA statement. Ann Intern Med.

[25] Musa B, Bussell S, Borodo MM, Samaila AA, Femi OL (2015). Prevalence of hepatitis B virus infection in Nigeria, 2000-2013: a systematic review and meta-analysis. Niger J Clin Pract.

[26] Downs SH, Black N (1998). The feasibility of creating a checklist for the assessment of the methodological quality both of randomized and non-randomized studies of health care interventions. J Epidemiol Community Health.

[27] Krajden M, McNabb G, Petric M (2005). The laboratory diagnosis of hepatitis B virus. Can J Infect Dis Med Microbiol.

[28] StatsDirect. Proportion Meta-analysis http://www.statsdirect.com/help/default.htm#meta_analysis/proportion.htm. Accessed 04 February 2016.

[29] Higgins JPT, Thompson SG, Deeks JJ, Altman DG (2003). Measuring inconsistency in meta-analyses. Br Med J.

[30] Addai-Mensah O, Bashiru PA, Dogbe EE (2015). Safety of family replacement donors vs. voluntary non-remunerated donors in Komfo Anokye Teaching Hospital, Ghana: a comparative study. J Med Biomed Sci.

[31] Adjei AA, Armah HB, Gbagbo F, Ampofo WK, Quaye IK, Hesse IF, Mensah G. Prevalence of human immunodeficiency virus, hepatitis B virus, hepatitis C virus and syphilis among prison inmates and officers at Nsawam and Accra, Ghana. J Med Microbiol. 2006;55(Pt 5):593–7. 10.1099/jmm.0.46414-0 16585647

[32] Adjei AA, Armah HB, Gbagbo F, Ampofo WK, Boamah I, Adu-Gyamfi C, Asare I, Hesse IF, Mensah G. Correlates of HIV, HBV, HCV and syphilis infections among prison inmates and officers in Ghana: a national multicenter study. BMC Infect Dis. 2008;8:33. 10.1186/1471-2334-8-33 PMC2311310 18328097

[33] Allain JP, Candotti D, Soldan K, Sarkodie F, Phelps B, Giachetti C, Shyamala V, et al. The risk of hepatitis B virus infection by transfusion in Kumasi, Ghana. Blood. 2003;101(6):2419–25. 10.1182/blood-2002-04-1084 12393691

[34] Allain JP, Opare-Sem O, Sarkodie F, Rahman R, Owusu-Ofori S (2009). Deferred donor care in a regional hospital blood center in Ghana. Transfusion.

[35] Allain JP, Sarkodie F, Asenso-Mensah K, Owusu-Ofori S (2010). Relative safety of first-time volunteer and replacement donors in West Africa. Transfusion.

[36] Amidu N, Owiredu WB, Addai-Mensah O, Alhassan A, Quaye L, Batong B (2010). Seroprevalence and risk factors for human immunodeficiency virus, hepatitis B and C Vi-ruses infections among blood donors at the Bolgatanga Regional Hospital in Bolgatanga, Ghana. J Ghana Sci Assoc.

[37] Amidu N, Alhassan A, Obirikorang C, Feglo P, Majeed SF, Timmy-Donkoh E, Afful D. Sero-prevalence of hepatitis B surface (HBsAg) antigen in three densely populated communities in Kumasi, Ghana. J Med Biomed Sci. 2012;1(2):59–65.

[38] Antwi-Baffour SS, Adarkwah-Yiadom K, Kyeremeh R, Adjei DN, Abdulai MS, Ayeh-Kumi PF (2014). Incidence of hepatitis B surface antigen among sickle cell disease patients receiving transfusion therapy. Int J Biomed Sci Engineering.

[39] Apea-Kubi KA, Yamaguchi S, Sakyi B (2006). Ofori-Adjei D.HTLV-1 and other viral sexually transmitted infections in antenatal and gynaecological patients in Ghana. West Afr J Med.

[40] Candotti D, Danso K, Allain JP (2007). Maternofetal transmission of hepatitis B virus genotype E in Ghana, West Africa. J Gen Virol.

[41] Cho Y, Bonsu G, Akoto-Ampaw A, Nkrumah-Mills G, Nimo JJ, Park JK, Ki M. The prevalence and risk factors for hepatitis B surface Ag positivity in pregnant women in eastern region of Ghana. Gut Liver. 2012;6(2):235–40. 10.5009/gnl.2012.6.2.235 PMC3343163 22570754

[42] Damale NK, Lassey AT, Bekoe V (2005). Hepatitis B virus seroprevalence among parturients in Accra, Ghana. Int J Gynecol Obstet.

[43] Dongdem JT, Kampo S, Soyiri IN, Asebga PN, Ziem JB, Sagoe K (2012). Prevalence of hepatitis B virus infection among blood donors at the Tamale Teaching Hospital, Ghana (2009). BMC Res Notes.

[44] Ephraim RK, Nsiah P, Osakunor DN, Adoba P, Sakyi SA, Anto EO (2014). Seroprevalence of hepatitis B and C viral infections among type 2 diabetics: a cross-sectional study in the Cape Coast Metropolis. Ann Med Health Sci Res.

[45] Ephraim R, Donkor I, Sakyi SA, Ampong J, Agbodjakey H (2015). Seroprevalence and risk factors of hepatitis B and hepatitis C infections among pregnant women in the Asante Akim North Municipality of the Ashanti region, Ghana; a cross sectional study. Afr Health Sci.

[46] Geretti AM, Patel M, Sarfo FS, Chadwick D, Verheyen J, Fraune M, Garcia A, Phillips RO. Detection of highly prevalent hepatitis B virus coinfection among HIV-seropositive persons in Ghana. J Clin Microbiol. 2010;48(9):3223–30. 10.1128/JCM.02231-09 PMC2937686 20631103

[47] Kubio C, Tierney G, Quaye T, Nabilsi JW, Ziemah C, Zagbeeb M, Shaw, Murphy WG. Blood transfusion practice in a rural hospital in Northern Ghana, Damongo, West Gonja District. Transfus Pract. 2012;52:2161–6. 10.1111/j.1537-2995.2012.03709.x 22612858

[48] Martinson FE, Weigle KA, Mushahwar IK, Weber DJ, Royce R, Lemon SM (1996). Seroepidemiological survey of hepatitis B and C virus infections in Ghanaian children. J Med Virol.

[49] Martinson FE, Weigle KA, Royce RA, Weber DJ, Suchindran CM, Lemon SM (1998). Risk factors for horizontal transmission of hepatitis B virus in a rural district in Ghana. Am J Epidemiol.

[50] Mutocheluh M, Owusu M, Kwofie TB, Akadigo T, Appau E, Narkwa PW (2014). Risk factors associated with hepatitis B exposure and the reliability of five rapid kits commonly used for screening blood donors in Ghana. BMC Res Notes.

[51] Nkrumah B, Owusu M, Frempong HO, Averu P (2011). Hepatitis B and C viral infections among blood donors from Rural Ghana. Ghana Med J.

[52] Nsiah K (2012). The prevalence of seropositivity to hepatitis B surface antigen and the corresponding hemato-biochemical features in sickle cell patients in Ghana. J Haematol Malig.

[53] Owiredu WK, Osei-Yeboah J, Amidu N, Laing EF (2012). Residual risk of transmission of hepatitis B virus through blood transfusion in Ghana: evaluation of the performance of rapid immunochromatographic assay with enzyme linked immunosorbent assay. J Med Biomed Sci.

[54] Owusu-Ofori S, Temple J, Sarkodie F, Anokwa M, Candotti D, Allain JP (2005). Predonation screening of blood donors with rapid tests: implementation and efficacy of a novel approach to blood safety in resource-poor settings. Transfusion.

[55] Rufai T, Mutocheluh M, Kwarteng K, Dogbe E (2014). The prevalence of hepatitis B virus E antigen among Ghanaian blood donors. Pan Afr Med J.

[56] Sagoe KW, Agyei AA, Ziga F, Lartey M, Adiku TK, Seshi M, Arens MQ, Mingle JA. Prevalence and impact of hepatitis B and C virus co-infections in antiretroviral treatment naïve patients with HIV infection at a major treatment center in Ghana. J Med Virol. 2012;84(1):6–10. 10.1002/jmv.22262 22095533

[57] Sarkodie F, Adarkwa M, Adu-Sarkodie Y, Candotti D, Acheampong JW (2001). Screening for viral markers in volunteer and replacement blood donors in West Africa. Vox Sang.

[58] Walana W, Hokey P, Ahiaba S (2014). Sero-prevalence of hepatitis B virus infection among blood donors: a retrospective study in the Kintampo Municipal hospital, Ghana. Open J Med Microbiol.

[59] Wasley A, Kruszon-Moran D, Kuhnert W, Simard EP, Finelli L, McQuillan G, Bell B. The prevalence of hepatitis B virus infection in the United States in the Era of vaccination. J Infect Dis. 2010;202(2):192–201. 10.1086/653622 20533878

[60] Alavian SM, Hajarizadeh B, Ahmadzad-Asl M, Kabir A, Bagheri-Lankarani K (2008). Hepatitis B virus infection in Iran: a systematic review. Hepatitis Monthly.

[61] Fejza H, Telaku S (2009). Prevalence of HBV and HCV among blood donors in Kosovo. Virol J.

[62] World Health Organization. Ghana National blood Policy. http://www.who.int/bloodsafety/transfusion_services/GhanaNationalBloodPolicy2006.pdf Accessed 10 January 2016.

[63] Mutocheluh M, Kwarteng K (2015). Knowledge and occupational hazards of barbers in the transmission of hepatitis B and C was low in Kumasi, Ghana. Pan Afr Med J.

[64] Akumiah PO, Sarfo LA (2015). Knowledge and practices of certified barbers about hepatitis B and C transmission in Kumasi, Ghana. Appl Res J.

[65] Lesi O. Hepatitis B in Africa: the challenges in controlling the scourge. http://theconversation.com/hepatitis-b-in-africa-the-challenges-in-controlling-the-scourge-43818 (2015). Accessed 02 Oct 2015

[66] Vildósola GH (2000). Hepatitis B, vaccination impact on acute disease, chronic carriers and hepatocarcinoma incidence. Rev Gastroenterol Peru.

[67] Menaca A, Tagbor H, Adjei R, Bart-Plange C, Collymore Y, Ba-Nguz A, Mertes K, Bingham A. Factors likely to affect community acceptance of a malaria vaccine in two Districts of Ghana: a qualitative study. PLoS One. 2014;9(10):e109707. 10.1371/journal.pone.0109707 PMC4198134 25334094

[68] World Hepatitis Alliance. Ghana Survey highlights. http://webcache.googleusercontent.com/search?q=cache:So4LDh_banUJ:global-report.worldhepatitisalliance.org/en/download/civil-society-download.html%3Ffile%3Dfiles/global_report/download/CS%2520countries/Ghana.pdf+&cd=1&hl=en&ct=clnk&gl=gh (2013). Accessed 01 Oct 2015.

[69] World Health Organization. Water Related Diseases. http://www.who.int/water_sanitation_health/diseases/hepatitis/en/(2015). Accessed 02 Oct 2015

[70] The World Bank Group. Improved water source (% of population with access). http://data.worldbank.org/indicator/SH.H2O.SAFE.ZS (2015). Accessed 03 Oct 2015.

[71] UNICEF. Ghana-WASH in Communities. http://www.unicef.org/ghana/wes.html (2013). Accessed 02 Oct 2015.

[72] Myjoyonline. Direct policy interventions towards treatment of Hepatitis B – Pharmacist http://lifestyle.myjoyonline.com/pages/health/201108/70392.php (2011). Accessed 01 October 2015.

[73] World Health Organization. World Health Assembly approves resolution on hepatitis and mechanism to coordinate non-communicable disease response. (2014) http://www.who.int/mediacentre/news/releases/2014/WHA-20140522/en/Accessed 03 Oct 2015.

[74] World Health Organization. Guidelines for the prevention, care and treatment of persons with chronic hepatitis B infection. http://www.who.int/hiv/pub/hepatitis/hepatitis-b-guidelines/en/(2015). Accessed 02 Oct 2015. 26225396

[75] Yohanes T, Zerdo Z, Chufamo N. “Seroprevalence and Predictors of Hepatitis B Virus Infection among Pregnant Women Attending Routine Antenatal Care in Arba Minch Hospital, South Ethiopia,” Hepatitis Research and Treatment. vol. 2016, Article ID 9290163, 7 pages, 2016. doi:10.1155/2016/9290163 10.1155/2016/9290163 PMC4745621 26904281

[76] Kuhns MC, Busch MP (2006). New strategies for blood donor screening for hepatitis B virus: nucleic acid testing versus immunoassay methods. Mol Diagn Ther.

